# Impact of GABA and nutritional supplements on neurochemical biomarkers in autism: a PPA rodent model study

**DOI:** 10.3389/fnmol.2025.1553438

**Published:** 2025-03-18

**Authors:** Altaf N. Alabdali, Abir Ben Bacha, Mona Alonazi, Sameera Abuaish, Ahmad Almotairi, Laila Al-Ayadhi, Afaf K. El-Ansary

**Affiliations:** ^1^Biochemistry Department, College of Sciences, King Saud University, Riyadh, Saudi Arabia; ^2^Department of Basic Sciences, College of Medicine, Princess Nourah Bint Abdulrahman University, Riyadh, Saudi Arabia; ^3^Department of Pathology, College of Medicine, King Saud University, Riyadh, Saudi Arabia; ^4^Autism Research and Treatment Center, Department of Physiology, Faculty of Medicine, King Saud University, Riyadh, Saudi Arabia; ^5^Autism Center, Lotus Holistic Medical Center, Abu Dhabi, United Arab Emirates

**Keywords:** autism spectrum disorder, excitatory-inhibitory balance, oxidative stress, EAAT2, KCC2, NKCC1, vitamin D3, GABRA5

## Abstract

**Background/objectives:**

Autism spectrum disorder (ASD) is associated with excitatory-inhibitory imbalance and oxidative stress. GABA, an inhibitory neurotransmitter, and related nutritional therapies are promising in restoring these imbalances. GABAergic deficits and glutamate excitotoxicity are two essential signaling pathways that could be addressed to treat autism, thus medications targeting these pathways are critical for treating behavioral symptoms. In a rat model of autism produced by propionic acid (PPA), this study assessed the effects of GABA supplementation and combined nutritional therapy (probiotics, vitamin D3) and *β*-lactam as an activator of glutamate transporter.

**Methods:**

Sixty rats were randomly assigned into six groups: Group I (Control), Group II (PPA-treated), Group III (Control-GABA), Group IV (Control-Combination), Group V (PPA-GABA), and Group VI (PPA-Combination). Social behavior was evaluated using the three-chamber test. Selected biochemical variables related to oxidative stress (GST, Catalase, Lipid peroxides, GSH and Vitamin C), GABA and glutamate signaling (EAAT2, KCC2, NKCC1, GABA, VD3, Glutamate and GABRA5) were measured in the brain homogenates of the six groups. The hippocampus was examined histopathologically to assess cellular integrity.

**Results:**

The obtained data revealed that PPA treatment caused significant oxidative stress and neurotransmitter imbalances, characterized by reduced GABA and elevated glutamate levels. GABA supplementation alone produced moderate benefits in biochemical and behavioral markers, but combined therapy considerably restored GABA levels, reduced oxidative stress, and enhanced social interaction behaviors. Histopathology revealed that combination therapy mitigated neurodegenerative changes induced by PPA, preserving hippocampal cellular structure.

**Conclusion:**

This study demonstrated that combined therapy (GABA, probiotics, vitamin D3, and *β*-lactam) were more effective than GABA alone in enhancing neurochemical balance and lowering oxidative stress in a PPA-induced mouse model of autism, indicating promise for treating symptoms.

## 1 Introduction

Autism Spectrum Disorder (ASD) is a complex neurodevelopmental condition characterized by deficits in social communication and the presence of restrictive and repetitive behaviors (American Psychiatric Association, 2013). While the exact cause of ASD remains elusive, it is widely accepted that both genetic and environmental factors contribute to its pathogenesis (Emberti Gialloreti et al., 2019). Globally, ASD affects approximately 1 in 100 children (World Health Organization, 2021), with a prevalence of 2.51% children, with a male to female ratio of 3:1in Riyadh, Saudi Arabia (AlBatti et al., 2022).

Although ASD is considered a common disorder, the exact etiology remains unknown (Sauer et al., 2021). Moreover, the current therapeutic approaches are severely constrained by a lack of understanding of the etiology and the correlations between the environment and behaviors. Animal models are crucial for the identification of the etiology of ASD and the development of treatment strategies, as it is virtually impossible to obtain brain tissue from living humans in daily practice and research. There have been numerous environmental agents that have been employed to develop animal models of ASDs (Lawler et al., 2004). Propionic acid (PPA) is a prospective agent that induces various behavioral and neuro-inflammatory alterations in the rat model of autism. PPA is a metabolic by product of intestinal bacteria and possesses the capability to cross the gut–blood and gut–brain barriers. Following transmission, PPA accumulates within the cells, resulting in intracellular acidification (Bonnet et al., 2000; El-Ansary et al., 2012). Thus, it has the potential to influence the normal functioning of a number of neurotransmitters (El-Ansary et al., 2012). At present, PPA is regarded as a well-established agent for the development of an experimental model of ASD (Sever et al., 2022; Sahin et al., 2022; Abujamel et al., 2022; Khera et al., 2022; Abuaish et al., 2022; Sharma et al., 2022).

The imbalance between neurotransmitters with excitatory and inhibitory properties is recognized as an etiological factor for ASD (Canitano and Palumbi, 2021). Gamma-aminobutyric acid (GABA) is a critical inhibitory neurotransmitter in the central nervous system (CNS). The inhibitory effect of GABAergic neurotransmission is important for the regulation of neurotransmission and the development of the brain. GABA has garnered significant attention in the investigation of the etiologies of a variety of neurological disorders, such as schizophrenia, depression, and anxiety (Amin et al., 2006; Möhler, 2012). Additionally, experimental studies have shown that core symptoms of ASD were substantially associated with dysfunction in the GABAergic signaling pathway (Cellot and Cherubini, 2014). The accumulating evidence indicated that the Na^+^–K^+^–2Cl^−^ cotransporter 1 (NKCC1) and K^+^–Cl^−^ cotransporter 2 (KCC2) were significantly correlated with GABAergic signaling (Kaila et al., 2014). The chloride importer NKCC1 and the chloride exporter KCC2 are critical components of GABA receptors (Lam et al., 2023).

Probiotics have the potential to reduce the symptoms of ASD by modulating neuroactive compounds. Additionally, emotional behavior and central GABA receptor expression were regulated by *Lactobacillus* treatment in a mouse through the vagus nerve, which serves as a communication pathway between the brain and the gut (Bravo et al., 2011). Probiotics may enhance inhibitory neurotransmission by upregulating GABA production or the expression of GABA receptor genes (El-Ansary et al., 2018; Bin-Khattaf et al., 2024). This may help to improve the social interaction deficits associated with ASD and restore the excitatory/inhibitory (E/I) balance.

Glutamate is an excitatory neurotransmitter in the brain. It is released from cells into the extracellular fluid and subsequently removed by glutamate transporters. Excitatory synaptic transmissions are regulated by this transportation. In autism, glutamate level is elevated, while glutamine levels are reduced (Ghanizadeh, 2013). One of the main glutamate transporters in the brain is the excitatory amino acid transporter EAAT2 (or GLT1). The EAAT2 variety comprises approximately 90% of glutamate transporters in the brain (Yamada et al., 2006). The extracellular glutamate level is maintained at a lower level than the neurotoxic level by excitatory amino acid transporters (Ghanizadeh and Berk, 2015). Glutamate neurotoxicity may serve as a pathological mechanism for autism (Harada et al., 2011). An imbalance of inhibitory and excitatory systems in the neurobiology of autism (Polleux and Lauder, 2004) is indicated by an abnormal GABA to glutamate ratio (Harada et al., 2011).

The *β*-lactam antibiotics cefixime and ceftriaxone are third-generation cephalosporins. GLT1/EAAT2 expression is increased by ceftriaxone (Nizzardo et al., 2011). Rothstein and colleagues demonstrated that many β-lactam antibiotics are able to increase EAAT2 protein levels through transcriptional activation (Rothstein et al., 2005). Ceftriaxone reduces extracellular glutamate levels by increasing the expression of astrocyte glutamate transporters (Zeng et al., 2010).

Growing evidence suggests that vitamin D likely participates in the pathogenesis of ASD, and vitamin D deficiency may be one of the causes of ASD (Mostafa and Al-Ayadhi, 2012; Gong et al., 2014; Fernell et al., 2015; Vinkhuyzen et al., 2018). Neurotransmitters associated with vitamin D are responsible for the regulation of learning, memory, and emotions (Patrick and Ames, 2014; Pertile et al., 2018; Anjum et al., 2018). Research indicates that prolonged administration of vitamin D to rodents can promote the synthesis of GABA production in brain regions, including the prefrontal cortex, anterior cingulate cortex, and hippocampus (Wang et al., 2022).

The purpose of this study is to evaluate the effectiveness of GABA and combined nutritional therapies as an intervention strategy for treating autism by employing a PPA-induced rodent model of autism. By measuring various biomarkers in brain tissue samples including GABA, glutamate, KCC2, KNCC1, Gamma-Aminobutyric Acid Receptor Subunit Alpha-5 (GABRA5), EAAT2, vitamin D3 (VD3) and several oxidative stress biomarkers. The study aimed to elucidate the neurochemical changes associated with different treatment regimens. Through this investigation, it is hoped to provide insights into how GABA supplementation and its combinations (*β*-lactam, probiotics and vitamin D3) affect the neurochemical pathways in ASD, thereby contributing to the development of more effective therapeutic strategies for the management of autism symptoms.

## 2 Materials and methods

### 2.1 Animals

Sixty young male western Albino rats, approximately 3 weeks old, weighing about 60–80 g, were obtained from the Experimental Surgery and Animal Laboratory at King Saud University (Riyadh, Saudi Arabia). Animals were housed in groups of 5 animals per cage at 22 ± 1°C and exposed to 12:12 h light–dark cycle and had access to food and water ad libitum. The Ethics Committee approved all procedures for Animal Research of King Saud University, Riyadh (IRB No: KSU-SE-23-58).

### 2.2 Experimental design

**Figure 1:**
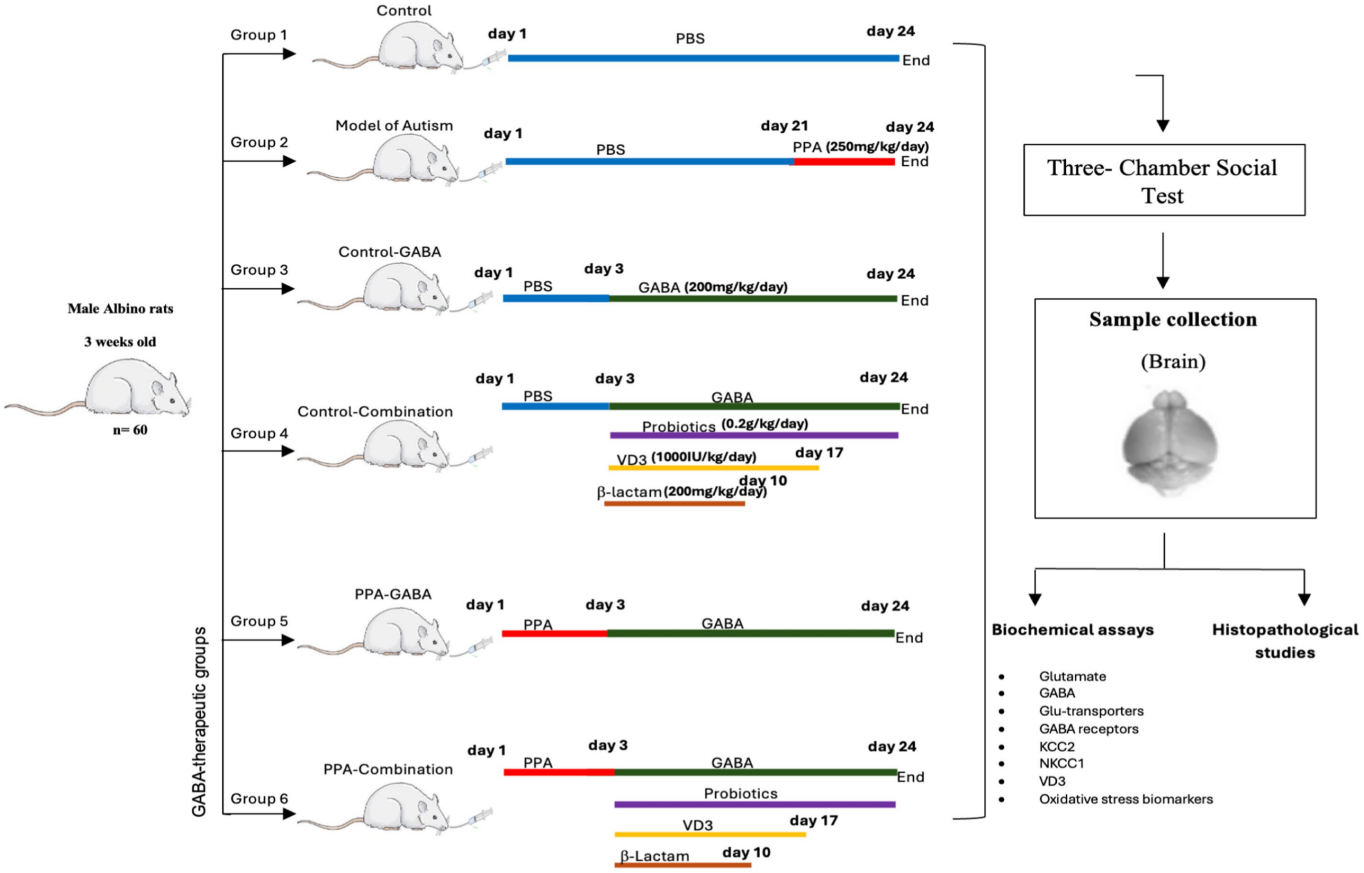
Diagrammatic scheme of the experimental design.

Sixty rats approximately 3 weeks old at the start of the treatment, were randomly assigned into six groups, with 10 rats in each group. Before the experiment began, all animals underwent a one-week acclimatization period to allow adaptation to the laboratory environment. Group I (Control) received phosphate-buffered saline (PBS) orally for 24 days. Group II (PPA group) received a neurotoxic dose of PPA (250 mg/kg body weight/day) orally via gavage for 3 days, followed by PBS for 21 days (El-Ansary et al., 2012). Group III (Control-GABA) received PBS for 3 days, then orally given GABA supplementation (ABA Pure powder, NOW®, 200 mg/kg body weight/day) for 21 days according to Abd El-Hady et al. (2017). Group IV (Control-combination) was administered PBS for 3 days, then orally given a combination therapy of GABA (200 mg/kg/day for 21 days), probiotics (Fiber probiotic, Proflora, 0.2 g/kg/day for 21 days), vitamin D3 (SUN-D 1000, California Greens, 1,000 IU/kg/day for 14 days) and *β*-lactam (Amoxicillin, HYMOX®, 200 mg/kg/day for 7 days) as described in previous studies (Bin-Khattaf et al., 2022; Alfawaz et al., 2014; Al-Suwailem et al., 2019). Group V (PPA-GABA), the GABA-therapeutic group, received the same oral PPA dose as Group II, then orally given GABA supplementation. Group VI (PPA-combination) was similarly orally administered PPA, then a combination therapy as described in Group IV (Figure 1).

### 2.3 Social interaction behavioral test

Social behavior was evaluated using the three-chamber social test, a widely used method to assess social deficits in animal models of autism (Schwartzer et al., 2013; Lo et al., 2016). The testing apparatus was a clear plexiglass box measuring 80 cm × 40 cm × 40 cm, divided into three chambers, each connected by a 15 cm × 15 cm doorway. Between trials, the three-chamberm box was cleaned with 70% ethanol, dried with paper towels, and left to air dry. To acclimate to the environment, animals were brought into the testing room 1 h before the test. Each animal was individually placed in the center chamber and allowed to explore freely for 5 min. After this habituation period, a novel, same-sex conspecific rat of similar body weight was placed in one of two perforated holding containers on either side of the box. The test subject was then given 10 min to explore all three chambers. To avoid side bias, the position of the conspecific rat was alternated between trials. The test was recorded using a Digital HERO camcorder (GoPro, San Mateo, California, United States). Recorded videos were later analyzed for behavior coding using BORIS 7.9.16 software (Friard and Gamba, 2016) by examiners blinded to the treatment groups. Social interaction time, defined as the test subject’s orientation toward and investigation of the container holding the conspecific rat through sniffing or rearing behaviors, was measured. Additionally, time spent in each chamber was recorded, and the percent of time spent in the social chamber (containing the conspecific rat) relative to all chamber was calculated. Finally, Immobility (bouts of no movement) and mobility time were also recorded.

### 2.4 Brain tissue collection and homogenization

At the end of the experiment, the rats were euthanized by inhalation of an overdose of anesthetic (sevoflurane). The brain was rapidly removed from the skull, flash-frozen on dry ice, and then stored at −80°C. Each brain was divided into two halves: one half was designated for biochemical assays, and the other for histopathological analysis. The half designated for biochemical assays was dissected into small pieces and completely homogenized in 10 w/v volumes of double-distilled water. Measurement of the selected biochemical markers, including oxidative stress biomarkers [glutathione-S-transferase (GST), catalase, lipid peroxide, glutathione (GSH) and vitamin C] and EAAT2, KCC2, KNCC1, GABA, VD3, glutamate and GABRA5 in the brain homogenates of the study groups was performed using a spectrophotometric and Enzyme-Linked Immunoassay (ELISA). The sample size for each biomarker was 46.

### 2.5 Biochemical analysis

#### 2.5.1 Oxidative stress biomarker assays

- GST: This enzyme was assessed by measuring the conjugation of 1-chloro,2,4-dinitrobenzene (CDNB) with GSH. This was followed by observing the increase in the OD at 340 nm.
- Catalase: The total volume of the reaction mixture was 3 mL. It contained 1.5 mL of 0.2 M sodium phosphate buffer pH 7.2, 1.2 mL of 0.5 mM hydrogen peroxide and enzyme. The reaction was started by adding hydrogen peroxide (H_2_O_2_), and the rate of change in absorbance was measured at 240 nm for 2 min (Aebi, 1984). Values were expressed as μmoles of H_2_O_2_ dissociated/min/dL brain homogenates, that is, U/dL.
- Lipid Peroxidation: Lipid oxidation was evaluated by measuring the levels of lipid peroxidation by-products as thiobarbituric acid reactive substances (TBARS), namely malondialdehyde (MD), using the method of Ruiz-Larrea et al. (1994). Accordingly, the samples were heated with TBA at low pH and the formation of a pink chromogen was measured by absorbance at 532 nm. The concentration of lipid peroxides was calculated as μmoles/ml using the extinction coefficient of MD.
- GSH: GSH content was determined according to the method described by Beutler et al. (1963) using 5,5′-dithiobis 2-nitrobenzoic acid (DTNB) with sulfhydryl compounds to produce a relatively stable yellow color.
- Vitamin C: Assay of vitamin C was performed according to the method of Jagota and Dani (1982). A quantity of 0.2 mL of brain homogenates was mixed with 0.8 mL of 10% trichloroacetic acid (TCA) and incubated in ice for 5 min. The samples were then centrifuged for 10 min at 3,500 rpm and 4°C. An amount of 1.5 mL double distilled water was subsequently added to 0.5 mL of the supernatant. Eventually, 2 mL of Folin-phenol reagent were added, and absorbance was measured at 760 nm after 10 min.

#### 2.5.2 Determination of EAAT2

The Rat EAAT2 ELISA kit (ELK7105, Wuhan, China) used a quantitative sandwich enzyme immunoassay. The microtiter plate was pre-coated with an EAAT2-specific antibody. After adding standards or samples and a biotin-conjugated EAAT2 antibody, avidin-HRP was added and incubated. The reaction was stopped with sulfuric acid, and the color change was measured at 450 nm. The detection range for EAAT2 was 0.16–10 ng/mL.

#### 2.5.3 Determination of KCC2

KCC2 levels were measured using an ELISA kit (ELK Biotechnology, ELK0481, Wuhan, China). The assay used a sandwich enzyme immunoassay with a microtiter plate pre-coated with an antibody specific to Rat KCC2. Standards or samples were added to the appropriate microtiter plate wells then with a biotin-conjugated KCC2 antibody. Next avidin-HRP were added. A substrate solution then produced a colorimetric reaction, with a detection range of 0.32–20 ng/mL.

#### 2.5.4 Determination of NKCC1

The Rat NKCC1 ELISA kit (ELK 0482, Wuhan, China) quantitatively determined NKCC1 concentrations in brain tissue using a sandwich enzyme immunoassay. The microtiter plate was pre-coated with an NKCC1-specific antibody. After adding standards or samples, a biotin-conjugated NKCC1 antibody and avidin-HRP were added. The reaction was stopped with sulfuric acid, and the color change was measured at 450 nm. The detection range was 0.32–20 ng/mL.

#### 2.5.5 Determination of GABA

The Rat GABA ELISA kit (ELK 9167, Wuhan, China) used a competitive inhibition enzyme immunoassay. The microtiter plate was pre-coated with GABA protein. After adding standards or samples and a biotin-conjugated GABA antibody, avidin-HRP was added and incubated. The reaction was stopped with sulfuric acid, and the color change was measured at 450 nm. The detection range was 31.25–2000 pg./mL.

#### 2.5.6 Determination of VD3

The Rat VD3 ELISA kit (ELK 8950, Wuhan, China) used a competitive inhibition enzyme immunoassay. The microtiter plate was pre-coated with VD3 protein. After adding standards or samples and a biotin-conjugated VD3 antibody, avidin-HRP was added and incubated. The reaction was stopped with sulfuric acid, and the color change was measured at 450 nm. The detection range was 6.25–400 ng/mL.

#### 2.5.7 Determination of glutamate

Glutamate levels in brain tissue were measured using a double-sandwich ELISA kit (MyBioSource Ltd., San Diego, CA, United States; Cat. No: MBS269969). The pre-coated antibody was an anti-Rat Glutamate monoclonal antibody, and the detecting antibody was biotinylated polyclonal antibody. After adding samples and biotinylated antibodies, Avidin-peroxidase conjugates were added. The reaction was measured at 450 nm, with a detection range of 0.312–20 nmoL/mL.

#### 2.5.8 Determination of GABRA5

GABRA5 levels in brain tissue were measured using a quantitative sandwich ELISA kit (MyBioSource Ltd., San Diego, CA, USA; Cat. No: MBS9335790). The microtiter plate was pre-coated with a GABRA5-specific antibody. The reaction was stopped with sulfuric acid, and the color change was measured at 450 nm. The detection range was 0.5–16 ng/mL.

### 2.6 Brain histology

Half of each rat’s brain (*n* = 12, with 2 brains from each experimental group) was fixed in 10% neutral buffered formalin. Then, they underwent routine histopathological processing, embedding in paraffin, sectioning to “5 μm thickness,” and staining with hematoxylin and eosin. The slides were then examined using light microscope by a certified surgical pathologist.

### 2.7 Statistical analysis

Statistical analysis was conducted using SPSS software (SPSS Inc., Chicago, IL, United States) version 22 for all analyses. Data were expressed as mean ± SD. One-way ANOVA and Kruskal-Wallis tests assessed group differences, followed by the Least Significant Difference (LSD) or Mann–Whitney tests for multiple comparisons. ROC curve analysis evaluated the diagnostic performance of biochemical parameters, reporting AUC, sensitivity, and specificity. Parameters with high AUC values were strong biomarker candidates. Stepwise multiple regression identified significant predictors, and standardized regression coefficients assessed their importance. Person correlation analysis explored relationships between biochemical parameters, reporting correlation coefficients (R) and *p* values. Animal behavior variables were analyzed by one- and two- way ANOVA followed by Tukey’s least significant difference test for multiple comparisons, with differences considered significant at *p* ≤ 0.05.

## 3 Results

### 3.1 Behavioral assessments

**Figure 2:**
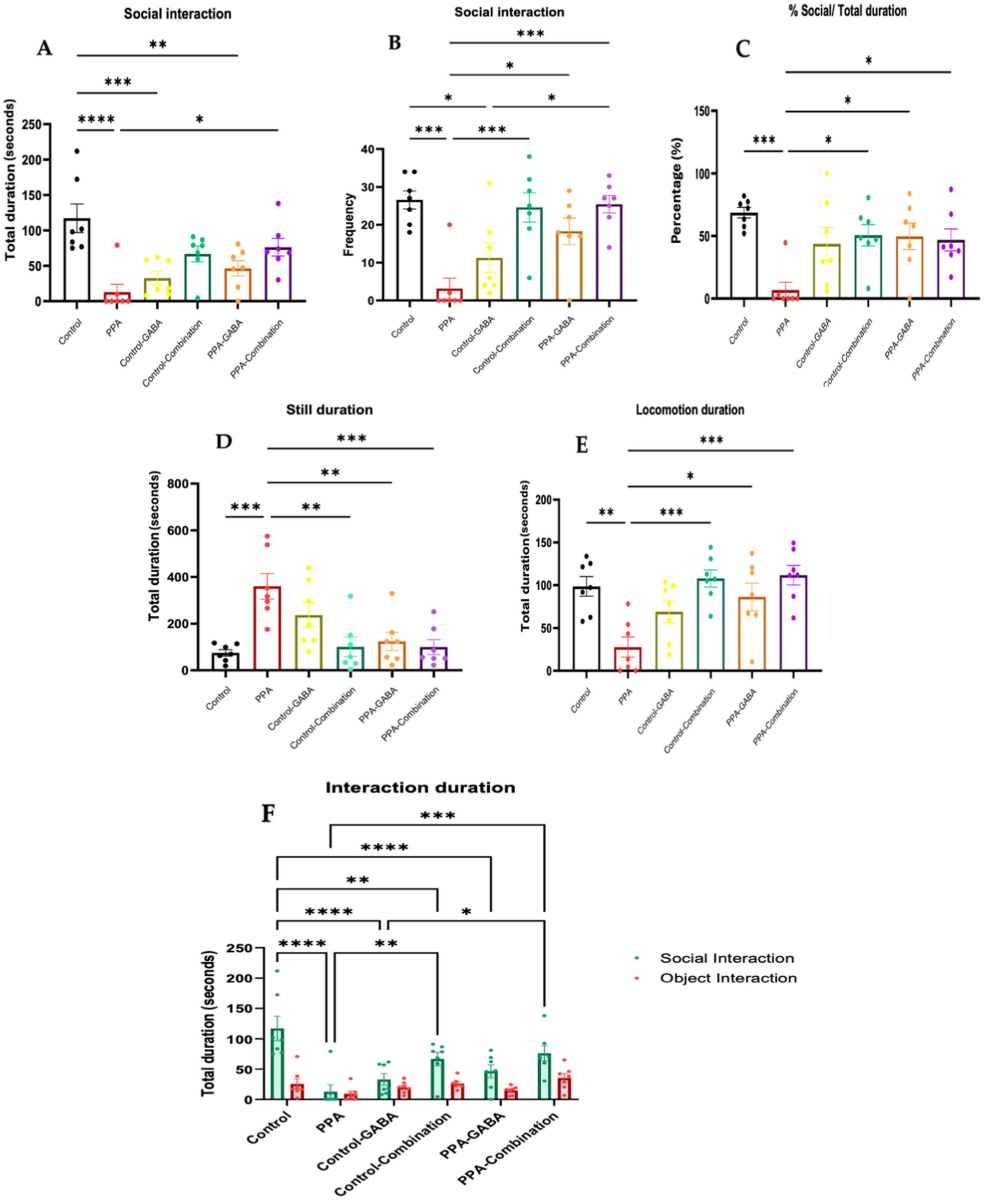
Social interaction in the three-chamber social test following control (*n* = 7), propionic acid (PPA) treatment, (*n* = 7), control-GABA (*n* = 7), control-combination interventions (*n* = 7), PPA-GABA (*n* = 7), PPA-combination (*n* = 7): **(A)** time spent (seconds) by focal animals interacting with the conspecific animal (social); **(B)** number of occurrences (count) by focal animals interacting with the conspecific animal (social); **(C)** percent of time (%) spent in the social chamber relative to other chambers; **(D)** time spent (seconds) immobile during the test; **(E)** time spent (seconds) mobile during the test; **(F)** time spent (seconds) by focal animals interacting with the conspecific animal (social) and the empty (object) holding box during the three-chamber social test. Data presented are means ± standard error. Significant one-way and two-way ANOVAs were followed by multiple comparisons by Tukey’s least significance difference ****p < 0.0001, ***p < 0.001, **p ≤ 0.01, *p ≤ 0.05.

The behavioral assessment shown in Figure 2 evaluated interactions in a three-chamber social test across the six groups: control, PPA-treated, control-GABA, control-combination, PPA-GABA, and PPA-combination. Results showed that PPA-treated animals spent significantly less time interacting with a conspecific animal than control animals (*p* < 0.0001; Figure 2A). GABA and combination therapies improved social interactions in PPA-treated animals, with the combination group showing the most significant recovery (*p* < 0.05 vs. PPA; Figure 2A). In terms of interaction frequency, control animals had higher interaction episodes than PPA-treated animals (*p* < 0.001; Figure 2B). Both PPA-GABA and PPA-combination groups improved interaction frequency, with the combination group showing the most significant improvement (*p* < 0.001; Figure 2B). Similarly, control animals spent more time in the social chamber than PPA-treated animals (*p* < 0.001; Figure 2C), but this deficit was alleviated by both PPA-GABA and PPA-combination therapies (*p* < 0.05 vs. PPA; Figure 2C). The PPA group spent more time immobile (*p* = 0.0003; Figure 2D), while both GABA and combination therapies reduced immobility, with the combination group showing the most improvement (*p* = 0.001; Figure 2D). Control animals displayed more locomotion than PPA-treated animals (*p* = 0.003; Figure 2E), whereas the combination therapy group showed the most significant recovery (*p* = 0.0004 vs. PPA; Figure 2E). Lastly, while control animals showed a preference for social interaction over object interaction, PPA-treated animals did not (Figure 2F). Remarkably, the combination therapy restored this preference, with the PPA-combination group spending significantly more time interacting with the conspecific animal (*p* = 0.0003 vs. PPA; Figure 2F). Notably, PPA-treated animals exhibited deficits in social interaction, locomotor activity, and social preference, while combination therapy provided the most significant recovery in all behavioral measures.

### 3.2 Biochemical analysis

The results from Table 1 and Supplementary Figure 1 demonstrate significant biochemical alterations across the different treatment groups. The PPA-treated group showed decreased antioxidant defenses, with reduced GST, catalase, GSH, and Vitamin C levels, alongside increased oxidative stress biomarkers such as lipid peroxides. The combination therapy (GABA, probiotics, VD3, and *β*-lactam) in PPA-combination group significantly restored these antioxidant biomarkers, particularly catalase and GSH levels. Additionally, neurotransmitter balance was disrupted in the PPA group, with lower GABA and higher glutamate levels. Both GABA and combination therapeutic groups improved GABA levels, with the PPA- combination group achieving the greatest recovery. The PPA- combination group also reduced glutamate levels, normalized EAAT2 function, and restored the GABA/Glutamate ratio, which was essential for improving inhibitory neurotransmission and restoring chloride cotransporters homeostasis (KCC2/NKCC1 ratio). One can see that PPA induced oxidative stress and neurotransmitter imbalances, while combination therapy significantly restored these markers.

**Table 1:** Comparison between different groups for the measured parameters.

Parameters	Groups	N	Min.	Max.	Mean ± S.D.	Median	Percent change (Mean)	*p* value
GST (μmol/ml)	Control group	8	0.11	0.22	0.17 ± 0.03	0.17	100.00	0.229
	PPA-Treated group	8	0.01	0.27	0.12 ± 0.07^f^	0.11	67.28	
	Control-GABA	9	0.05	0.21	0.13 ± 0.05	0.13	73.98	
	Control-Combination	7	0.09	0.18	0.15 ± 0.03	0.15	85.57	
	PPA-GABA	7	0.04	0.35	0.16 ± 0.09	0.15	92.50	
	PPA-Combination	7	0.14	0.25	0.18 ± 0.04	0.18	103.64	
Catalase (μmol/ml)	Control group	8	0.14	0.55	0.28 ± 0.13 ^c^	0.28	100.00	0.029
	PPA-Treated group	8	0.07	0.28	0.17 ± 0.07^c^^,^^d^^,^^e^^,^^f^	0.16	61.14	
	Control-GABA	9	0.07	1.65	0.71 ± 0.45^a^^,^^b^	0.71	253.33	
	Control-Combination	7	0.05	2.04	0.66 ± 0.70^b^	0.34	233.51	
	PPA-GABA	7	0.34	0.99	0.59 ± 0.20^b^	0.57	208.95	
	PPA-Combination	7	0.05	1.28	0.68 ± 0.41^b^	0.67	241.68	
Lipid peroxides (μmol/ml)	Control group	8	1.45	2.79	2.19 ± 0.46	2.26	100.00	0.098
	PPA-Treated group	8	1.65	3.59	2.37 ± 0.63	2.41	108.28	
	Control-GABA	9	1.20	2.95	1.97 ± 0.69	1.81	89.85	
	Control-Combination	7	1.09	2.09	1.53 ± 0.35	1.59	69.86	
	PPA-GABA	7	1.56	2.65	2.18 ± 0.42	2.23	99.41	
	PPA-Combination	7	1.23	2.90	2.09 ± 0.62	2.00	95.63	
GSH (μg/ml)	Control group	8	2.77	11.54	6.55 ± 2.81^b^^,^^c^^,^^f^	6.92	100.00	0.001
	PPA-Treated group	8	−0.25	8.25	2.67 ± 3.23^a^^,^^c^^,^^d^^,^^f^	1.67	40.73	
	Control-GABA	9	6.66	18.24	9.39 ± 3.53^a^^,^^b^^,^^d^^,^^e^^,^^f^	8.25	143.38	
	Control-Combination	7	2.77	8.94	5.85 ± 2.31^b^^,^^c^^,^^f^	6.06	89.25	
	PPA-GABA	7	2.09	7.16	4.30 ± 1.91^c^^,^^f^	4.01	65.62	
	PPA-Combination	7	11.54	14.82	13.41 ± 1.36^a^^,^^b^^,^^c^^,^^d^^,^^e^	13.73	204.79	
VitC (μg/ml)	Control group	8	2.54	10.81	6.81 ± 3.61^d^	7.29	100.00	0.007
	PPA-Treated group	8	2.54	9.29	6.14 ± 2.73^d^^,^^e^^,^^f^	7.36	90.14	
	Control-GABA	9	2.54	14.94	8.04 ± 4.24^d^	8.05	117.98	
	Control-Combination	7	8.68	16.32	12.27 ± 3.15^a^^,^^b^^,^^c^	12.18	180.19	
	PPA-GABA	7	8.05	13.56	9.63 ± 2.31^b^	8.05	141.32	
	PPA-Combination	7	8.05	14.94	10.00 ± 2.37^b^	9.43	146.80	
EAAT2 (ng/ml)	Control group	8	2.26	11.71	6.87 ± 3.52^c^^,^^e^^,^^f^	5.74	100.00	0.001
	PPA-Treated group	8	1.49	9.48	4.34 ± 2.54^c^^,^^d^^,^^f^	4.17	63.23	
	Control-GABA	9	0.92	4.08	1.72 ± 1.03^a^^,^^b^^,^^d^	1.46	25.10	
	Control-Combination	7	3.39	12.26	7.93 ± 3.53^b^^,^^c^^,^^e^^,^^f^	7.86	115.47	
	PPA-GABA	7	1.02	8.96	3.03 ± 2.87^a^^,^^d^	1.74	44.12	
	PPA-Combination	7	0.82	1.92	1.20 ± 0.40^a^^,^^b^^,^^d^	1.00	17.51	
KCC2 (ng/ml)	Control group	8	12.14	20.17	16.26 ± 2.51	16.57	100.00	0.341
	PPA-Treated group	8	14.90	26.56	22.58 ± 4.31	24.24	138.88	
	Control-GABA	9	9.42	30.67	19.61 ± 7.26	18.03	120.64	
	Control-Combination	7	16.42	27.00	20.52 ± 4.13	18.44	126.24	
	PPA-GABA	7	10.85	30.21	19.55 ± 7.57	16.94	120.26	
	PPA-Combination	7	13.02	26.54	20.70 ± 4.74	20.28	127.35	
NKCC1 (ng/ml)	Control group	8	9.45	15.81	12.11 ± 2.30	11.91	100.00	0.020
	PPA-Treated group	8	9.90	24.14	16.05 ± 4.62^c^^,^^f^	15.83	132.54	
	Control-GABA	9	6.12	17.86	10.60 ± 4.28^b^	10.27	87.49	
	Control-Combination	7	6.14	23.42	14.93 ± 6.48^f^	13.88	123.27	
	PPA-GABA	7	5.12	22.71	11.77 ± 7.44	7.96	97.20	
	PPA-Combination	7	4.16	13.44	7.11 ± 3.43^b^^,^^d^	5.83	58.72	
GABA (ng/ml)	Control group	8	0.016	0.049	0.033 ± 0.009^c^^,^^d^^,^^e^^,^^f^	0.033	100.00	0.001
	PPA-Treated group	8	0.011	0.045	0.022 ± 0.011^c^^,^^d^^,^^e^^,^^f^	0.019	66.38	
	Control-GABA	9	0.016	0.099	0.056 ± 0.029^a^^,^^b^^,^^e^^,^^f^	0.061	168.17	
	Control-Combination	7	0.035	0.097	0.059 ± 0.020^a^^,^^b^^,^^e^^,^^f^	0.058	175.45	
	PPA-GABA	7	0.051	0.121	0.092 ± 0.023^a^^,^^b^^,^^c^^,^^d^	0.096	272.98	
	PPA-Combination	7	0.088	0.133	0.112 ± 0.013^a^^,^^b^^,^^c^^,^^d^	0.111	331.01	
VD3 (ng/ml)	Control group	8	1.70	2.81	2.16 ± 0.37	2.06	100.00	0.361
	PPA-Treated group	8	2.07	2.71	2.26 ± 0.25	2.11	104.95	
	Control-GABA	9	1.71	2.74	2.30 ± 0.32	2.31	106.87	
	Control-Combination	7	1.95	2.98	2.29 ± 0.38	2.13	106.09	
	PPA-GABA	7	1.76	3.79	2.71 ± 0.80	2.36	125.76	
	PPA-Combination	7	1.83	3.61	2.36 ± 0.63	2.21	109.58	
Glutamate (ng/ml)	Control group	8	1.00E+05	1.30E+05	1.16E+05 ± 8.36E+03^f^	1.17E+05	100.00	0.003
	PPA-Treated group	8	1.08E+05	1.34E+05	1.22E+05 ± 1.08E+04^e^^,^^f^	1.23E+05	105.05	
	Control-GABA	9	9.86E+04	1.56E+05	1.21E+05 ± 2.12E+04^f^	1.12E+05	103.92	
	Control-Combination	7	9.24E+04	1.35E+05	1.14E+05 ± 1.42E+04^f^	1.15E+05	98.17	
	PPA-GABA	7	8.68E+04	1.41E+05	1.06E+05 ± 1.89E+04^b^	1.01E+05	91.54	
	PPA-Combination	7	7.97E+04	1.09E+05	9.16E+04 ± 1.14E+04^a^^,^^b^^,^^c^^,^^d^	8.84E+04	78.80	
GABRA5 (ng/ml)	Control group	8	0.45	0.74	0.58 ± 0.11	0.59	100.00	0.959
	PPA-Treated group	8	0.09	1.01	0.58 ± 0.34	0.68	99.67	
	Control-GABA	9	0.09	1.37	0.58 ± 0.36	0.51	100.00	
	Control-Combination	7	0.18	1.03	0.49 ± 0.29	0.49	84.20	
	PPA-GABA	7	0.35	1.06	0.63 ± 0.24	0.55	107.90	
	PPA-Combination	7	0.34	1.20	0.62 ± 0.31	0.52	106.39	
KCC2/NKCC1	Control group	8	0.85	1.71	1.38 ± 0.31^f^	1.37	100.00	0.001
	PPA-Treated group	8	0.70	2.61	1.55 ± 0.62^f^	1.56	112.33	
	Control-GABA	9	0.72	3.67	2.02 ± 0.83^f^	1.95	146.34	
	Control-Combination	7	0.79	2.97	1.64 ± 0.85^f^	1.25	119.00	
	PPA-GABA	7	0.59	3.07	2.05 ± 0.91^f^	2.13	148.63	
	PPA-Combination	7	1.97	4.41	3.26 ± 1.10^a^^,^^b^^,^^c^^,^^d^^,^^e^	3.59	236.29	
GABA/Glutamate	Control group	8	1.37E-07	4.40E-07	2.94E-07 ± 8.78E-08^e^^,^^f^	2.85E-07	100.00	0.001
	PPA-Treated group	8	8.93E-08	4.11E-07	1.89E-07 ± 1.06E-07^c^^,^^d^^,^^e^^,^^f^	1.61E-07	64.34	
	Control-GABA	9	1.09E-07	9.48E-07	5.16E-07 ± 3.08E-07^b^^,^^e^^,^^f^	6.25E-07	175.35	
	Control-Combination	7	2.94E-07	1.06E-06	5.43E-07 ± 2.56E-07^b^^,^^e^^,^^f^	4.32E-07	184.67	
	PPA-GABA	7	3.63E-07	1.40E-06	9.13E-07 ± 3.28E-07^a^^,^^b^^,^^c^^,^^d^^,^^f^	9.44E-07	310.29	
	PPA-Combination	7	8.08E-07	1.63E-06	1.25E-06 ± 2.67E-07^a^^,^^b^^,^^c^^,^^d^^,^^e^	1.25E-06	425.02	

The table describes one-way ANOVA Test between different groups with multiple comparisons (LSD test) within the entire groups for each parameter.

a Describes significant difference between the group and the (Control group) at significant level (0.05).

b Describes significant difference between the group and the (PPA-Treated group) at significant level (0.05).

c Describes significant difference between the group and the (Control-GABA) at significant level (0.05).

d Describes significant difference between the group and the (Control-Combination) at significant level (0.05).

e Describes significant difference between the group and the (PPA-GABA) at significant level (0.05).

f Describes significant difference between the group and the (PPA-Combination) at significant level (0.05).

### 3.3 Correlation analysis

The results in Table 2 revealed significant correlations between various oxidative stress biomarkers and neurotransmitters system. There was a positive correlation between catalase and GSH (*r* = 0.311, *p* = 0.035), and between GSH with GABA (*r* = 0.401, *p* = 0.006) and GABA/ Glutamate (*r* = 0.432, *p* = 0.003). Vitamin C is positively correlated with GABA (*r* = 0.305, *p* = 0.039), and the KCC2/NKCC1 ratio positively correlates with the GABA/Glutamate ratio (*r* = 0.718, *p* = 0.000). Additionally, VD3 positively correlated with GABA and the GABA/Glutamate ratio. In contrast, negative correlations were observed between lipid peroxides and Vitamin C (*r* = −0.318, *p* = 0.031), lipid peroxides with EAAT2 (*r* = −0.295, *p* = 0.047) and KCC2 (*r* = −0.344, *p* = 0.019), and NKCC1 and GABA (*r* = −0.622, *p* = 0.000). Additionally, GSH negatively correlated with EAAT2 (*r* = −0.349, *p* = 0.017) and NKCC1 (*r* = −0.376, *p* = 0.010) and Glutamate (*r* = −0.325, *p* = 0.027). Lastly, Glutamate negatively correlated with GABA (*r* = −0.771, *p* = 0.000) and the KCC2/NKCC1 ratio (*r* = −0.609, *p* = 0.000). Thus, GABA levels were strongly correlated with antioxidant status, while oxidative stress negatively impacted neurotransmitter function.

**Table 2:** Correlations between the measured parameters using Person correlation.

Parameters	R (Correlation coefficient)	*p* value
Catalase–GSH	0.311*	0.035
Lipid peroxides–VitC	−0.318*	0.031
Lipid peroxides–EAAT2	−0.295*	0.047
Lipid peroxides–KCC2	−0.344*	0.019
GSH–EAAT2	−0.349*	0.017
GSH–NKCC1	−0.376**	0.010
GSH–GABA	0.401**	0.006
GSH–Glutamate	−0.325*	0.027
GSH–KCC2/NKCC1	0.350*	0.017
GSH–GABA/Glutamate	0.432**	0.003
VitC–GABA	0.305*	0.039
EAAT2–GABA	−0.355*	0.015
EAAT2–KCC2/NKCC1	−0.372*	0.011
EAAT2–GABA/Glutamate	−0.373*	0.011
KCC2–VD3	−0.395**	0.007
KCC2–Glutamate	0.324*	0.028
KCC2–GABRA5	0.323*	0.029
NKCC1–GABA	−0.622**	0.000
NKCC1–VD3	−0.378**	0.010
NKCC1–Glutamate	0.691**	0.000
NKCC1–KCC2/NKCC1	−0.761**	0.000
NKCC1–GABA/Glutamate	−0.674**	0.000
GABA–VD3	0.422**	0.004
GABA–Glutamate	−0.771**	0.000
GABA–KCC2/NKCC1	0.658**	0.000
GABA–GABA/Glutamate	0.982**	0.000
VD3–Glutamate	−0.424**	0.003
VD3–GABA/Glutamate	0.459**	0.001
Glutamate–GABRA5	0.299*	0.043
Glutamate–KCC2/NKCC1	−0.609**	0.000
Glutamate–GABA/Glutamate	−0.832**	0.000
KCC2/NKCC1–GABA/Glutamate	0.718**	0.000

*Correlation is significant at the 0.05 level. **Correlation is significant at the 0.01 level.

### 3.4 Multiple regression analysis

The results from the multiple regression analyses across Tables 3–15 highlighted key predictors influencing various biomarkers. Table 3 indicated that GSH significantly predicted catalase levels (*p* = 0.035), explaining around 7.6% of the variance. Table 4 identified KCC2, vitamin C, and EAAT2 as significant negative predictors of lipid peroxides, explaining up to 25.5% of the variance. In Table 5, the GABA/Glutamate ratio and VD3 significantly predicted GSH levels (*p* = 0.003 and *p* = 0.013, respectively), with the GABA/Glutamate ratio being the strongest predictor. In Table 6, lipid peroxides negatively predicted vitamin C levels (*p* = 0.023), while GABA positively influenced vitamin C. Table 7 showed that the GABA/Glutamate ratio and lipid peroxides negatively predicted EAAT2 levels, with glutamate and KCC2/NKCC1 also having negative influences. Table 8 showed that VD3 and the KCC2/NKCC1 ratio significantly predicted KCC2 levels. VD3 having a negative relationship, while KCC2/NKCC1 positively influenced KCC2. Table 9 revealed that KCC2/NKCC1 was the strongest predictor of NKCC1 levels. Table 10 showed that the GABA/Glutamate ratio and Glutamate significantly predicted GABA levels, with a very high adjusted R-square (0.973). Table 11 highlighted the GABA/Glutamate ratio and GSH as significant predictors of VD3 levels, where the GABA/Glutamate ratio positively correlated with VD3, while GSH showed a negative association. Table 12 demonstrated that the GABA/Glutamate ratio and GABA were strong predictors of Glutamate levels, with the GABA/Glutamate ratio negatively correlating with Glutamate. Table 13 revealed that KCC2 was a significant predictor for GABRA5, showing a positive correlation. Table 14 indicated that NKCC1, KCC2 and GABA/Glutamate ratio were potent predictors for the KCC2/NKCC1 ratio. Table 15 identified GABA and Glutamate as key predictors of the GABA/Glutamate ratio, with GABA positively influencing the ratio and Glutamate having a negative effect. Overall, neurotransmitter imbalances and oxidative stress markers were highly predictive of biochemical changes.

**Table 3:** Multiple regression using stepwise method for catalase as a dependent variable.

Predictor variable	Coefficient	S.E.	*p* value	Adjusted R square	95% CI	
					Lower	Upper
GSH	0.030	0.014	0.035	0.076	0.002	0.059

**Table 4:** Multiple regression using stepwise method for lipid peroxides as a dependent variable.

Predictor variable	Coefficient	S.E.	*p* value	Adjusted R square	95% CI	
					Lower	Upper
KCC2	−0.037	0.015	0.019	0.099	−0.067	−0.006
KCC2	−0.038	0.014	0.012	0.190	−0.066	−0.009
VitC	−0.052	0.021	0.019		−0.095	−0.009
KCC2	−0.037	0.014	0.009	0.255	−0.065	−0.010
VitC	−0.050	0.020	0.018		−0.092	−0.009
EAAT2	−0.047	0.021	0.035		−0.090	−0.003

**Table 5:** Multiple regression using stepwise method for GSH as a dependent variable.

Predictor variable	Coefficient	S.E.	*p* value	Adjusted R square	95% CI	
					Lower	Upper
GABA/Glutamate	4380.631	1380.282	0.003	0.168	1598.856	7162.406
GABA/Glutamate	6124.475	1460.700	0.000	0.264	3178.692	9070.258
VD3	−3.331	1.280	0.013		−5.913	−0.749

**Table 6:** Multiple regression using stepwise method for VitC as a dependent variable.

Predictor variable	Coefficient	S.E.	*p* value	Adjusted R square	95% CI	
					Lower	Upper
Lipid peroxides	−2.005	0.901	0.031	0.081	−3.820	−0.190
Lipid peroxides	−2.028	0.861	0.023	0.159	−3.765	−0.291
GABA	0.032	0.014	0.029		0.003	0.060

**Table 7:** Multiple regression using stepwise method for EAAT2 as a dependent variable.

Predictor variable	Coefficient	S.E.	*p* value	Adjusted R square	95% CI	
					Lower	Upper
GABA/Glutamate	−3046.228	1143.254	0.011	0.119	−5350.305	−742.152
GABA/Glutamate	−2940.346	1104.560	0.011	0.180	−5167.904	−712.788
Lipid peroxides	−1.668	0.810	0.046		−3.302	−0.034
GABA/Glutamate	−6670.081	1940.037	0.001	0.253	−10585.234	−2754.929
Lipid peroxides	−2.174	0.804	0.010		−3.797	−0.552
Glutamate	−1.10E-04	4.80E-05	0.027		−2.07E-04	−1.30E-05
GABA/Glutamate	−4771.982	2091.915	0.028	0.305	−8996.689	−547.274
Lipid peroxides	−2.585	0.802	0.002		−4.204	−0.966
Glutamate	−1.20E-04	4.70E-05	0.014		−2.14E-04	−2.50E-05
KCC2/NKCC1	−1.353	0.666	0.049		−2.698	−0.008

**Table 8:** Multiple regression using stepwise method for KCC2 as a dependent variable.

Predictor variable	Coefficient	S.E.	*p* value	Adjusted R square	95% CI	
					Lower	Upper
VD3	−4.440	1.558	0.007	0.137	−7.579	−1.301
VD3	−5.363	1.485	0.001	0.253	−8.358	−2.367
KCC2/NKCC1	2.101	0.749	0.007		0.592	3.611
VD3	−3.144	1.212	0.013	0.558	−5.589	−0.698
KCC2/NKCC1	5.711	0.871	0.000		3.954	7.468
NKCC1	0.890	0.161	0.000		0.565	1.215
VD3	−2.147	1.135	0.066	0.640	−4.438	0.145
KCC2/NKCC1	6.266	0.803	0.000		4.643	7.888
NKCC1	0.705	0.156	0.000		0.390	1.020
Glutamate	1.33E-04	4.10E-05	0.002		5.10E-05	2.15E-04
VD3	−2.887	1.144	0.016	0.669	−5.198	−0.575
KCC2/NKCC1	6.335	0.772	0.000		4.775	7.895
NKCC1	0.642	0.152	0.000		0.334	0.951
Glutamate	1.19E-04	4.00E-05	0.005		3.90E-05	1.99E-04
GSH	−0.263	0.124	0.040		−0.514	−0.013

**Table 9:** Multiple regression using stepwise method for NKCC1 as a dependent variable.

Predictor Variable	Coefficient	S.E.	*p* value	Adjusted R square	95% CI	
					Lower	Upper
KCC2/NKCC1	−4.333	0.558	0.000	0.569	−5.458	−3.209
KCC2/NKCC1	−5.042	0.420	0.000	0.773	−5.889	−4.195
KCC2	0.471	0.074	0.000		0.322	0.621

**Table 10:** Multiple regression using stepwise method for GABA as a dependent variable.

Predictor variable	Coefficient	S.E.	*p* value	Adjusted R square	95% CI	
					Lower	Upper
GABA/Glutamate	8.27E+04	2412.41	0.000	0.963	7.78E+04	8.75E+04
GABA/Glutamate	9.31E+04	3959.732	0.000	0.969	8.52E+04	1.01E+05
Glutamate	3.06E-04	9.60E-05	0.003		1.12E-04	5.01E-04
GABA/Glutamate	9.85E+04	4227.887	0.000	0.973	9.00E+04	1.07E+05
Glutamate	2.99E-04	9.00E-05	0.002		1.17E-04	4.82E-04
KCC2/NKCC1	−3.453	1.305	0.011		−6.087	−0.820

**Table 11:** Multiple regression using stepwise method for VD3 as a dependent variable.

Predictor Variable	Coefficient	S.E.	*p* value	Adjusted R square	95% CI	
					Lower	Upper
GABA/Glutamate	523.553	152.823	0.001	0.193	215.559	831.548
GABA/Glutamate	702.418	159.294	0.000	0.286	381.171	1023.666
GSH	−4.10E-02	1.60E-02	0.013		−7.20E-02	−9.00E-03
GABA/Glutamate	651.167	146.480	0.000	0.404	355.559	946.776
GSH	−4.50E-02	1.40E-02	0.003		−7.40E-02	−1.60E-02
KCC2	−0.032	0.010	0.004		−0.053	−0.011

**Table 12:** Multiple regression using stepwise method for glutamate as a dependent variable.

Predictor variable	Coefficient	S.E.	*p* value	Adjusted R square	95% CI	
					Lower	Upper
GABA/Glutamate	−3.42E+07	3.44E+06	0.000	0.686	−4.11E+07	−2.73E+07
GABA/Glutamate	−8.56E+07	1.65E+07	0.000	0.739	−1.19E+08	−5.24E+07
GABA	621.290	195.402	0.003		227.223	1015.356
GABA/Glutamate	−8.19E+07	1.57E+07	0.000	0.764	−1.14E+08	−5.02E+07
GABA	591.911	186.453	0.003		215.634	968.188
KCC2	550.553	235.972	0.025		74.342	1026.765
GABA/Glutamate	−7.89E+07	1.52E+07	0.000	0.783	−1.10E+08	−4.83E+07
GABA	539.030	180.486	0.005		174.532	903.529
KCC2	545.397	226.321	0.021		88.332	1002.462
Catalase	6439.261	2981.814	0.037		417.365	12461.157

**Table 13:** Multiple regression using stepwise method for GABRA5 (ng/ml) as a dependent variable.

Predictor variable	Coefficient	S.E.	*p* value	Adjusted R square	95% CI	
					Lower	Upper
KCC2	0.016	0.007	0.029	0.084	0.002	0.031

**Table 14:** Multiple regression using stepwise method for KCC2/NKCC1 as a dependent variable.

Predictor variable	Coefficient	S.E.	*p* value	Adjusted R square	95% CI	
					Lower	Upper
NKCC1	−0.133	0.017	0.000	0.569	−0.168	−0.099
NKCC1	−0.153	0.013	0.000	0.776	−0.178	−0.127
KCC2	0.083	0.013	0.000		0.057	0.109
NKCC1	−0.107	0.014	0.000	0.858	−0.135	−0.080
KCC2	0.084	0.010	0.000		0.063	0.104
GABA/Glutamate	873.739	171.984	0.000		526.661	1220.818
NKCC1	−0.097	0.013	0.000	0.882	−0.122	−0.071
KCC2	0.084	0.009	0.000		0.066	0.103
GABA/Glutamate	2892.075	672.400	0.000		1534.135	4250.015
GABA	−0.023	0.008	0.004		−0.038	−0.008

**Table 15:** Multiple regression using stepwise method for GABA/Glutamate as a dependent variable.

Predictor variable	Coefficient	S.E.	*p* value	Adjusted R square	95% CI	
					Lower	Upper
GABA	1.17E-05	3.40E-07	0.000	0.963	1.10E-05	1.24E-05
GABA	9.96E-06	4.24E-07	0.000	0.977	9.11E-06	1.08E-05
Glutamate	−4.51E-09	8.68E-10	0.000		−6.26E-09	−2.76E-09
GABA	9.42E-06	4.04E-07	0.000	0.982	8.61E-06	1.02E-05
Glutamate	−3.92E-09	7.87E-10	0.000		−5.51E-09	−2.33E-09
KCC2/NKCC1	4.32E-05	1.21E-05	0.001		1.88E-05	6.75E-05

### 3.5 ROC analysis

The ROC analysis (Tables 16–20; Supplementary Figures 2–6) evaluated the diagnostic ability of various biomarkers to differentiate between treatment groups (PPA-treated, control-GABA, control-combination, PPA-GABA and PPA-combination) using the control group as a reference. The Area Under the Curve (AUC) values give insight into the accuracy of these biomarkers. In Table 16 and Supplementary Figure 2 (PPA-treated vs. control), GST, GSH, KCC2 and GABA showed relatively high AUC values (AUC > 0.75), with high sensitivity and specificity. For example, KCC2 showed good diagnostic accuracy (AUC = 0.844) with 75% sensitivity and 100% specificity. Table 17 and Supplementary Figure 3 (Control-GABA vs. control) highlighted catalase (AUC = 0.847, *p* = 0.016) and EAAT2 (AUC = 0.958, *p* = 0.001) as good predictors, while other parameters showed moderate diagnostic value. Table 18 and Supplementary Figure 4 (Control-Combination vs. control), GABA again showed strong diagnostic potential (AUC = 0.920), as did lipid peroxides and GABA/Glutamate ratio. Table 19 and Supplementary Figure 5 (PPA-GABA vs. control) demonstrated GABA and the GABA/Glutamate ratio as strong predictors (AUC = 1.000 for both), with high sensitivity and specificity. Likewise, Table 20 and Supplementary Figure 6 (PPA-Combination vs. control) revealed several parameters, including GABA, GSH, EAAT2, and KCC2/NKCC1 and GABA/Glutamate ratio, with AUC values of 1.000. Therefore, GABA, EAAT2, and KCC2 were the most reliable biomarkers for distinguishing treatment effects.

**Table 16:** ROC analysis of the measured parameters in PPA-Treated group.

Parameters	AUC	Cut-off value	Sensitivity %	Specificity %	*p* value	95% CI
GST	0.813	0.151	87.5%	87.5%	0.036	0.564–1.000
Catalase	0.766	0.183	75.0%	75.0%	0.074	0.527–1.000
Lipid peroxides	0.586	2.300	62.5%	62.5%	0.564	0.295–0.876
GSH	0.797	4.623	75.0%	75.0%	0.046	0.565–1.000
VitC	0.602	8.671	87.5%	50.0%	0.495	0.304–0.899
EAAT2	0.750	4.841	75.0%	75.0%	0.093	0.504–0.996
KCC2	0.844	21.384	75.0%	100.0%	0.021	0.632–1.000
NKCC1	0.781	13.500	75.0%	75.0%	0.059	0.546–1.000
GABA	0.797	32.707	87.5%	75.0%	0.046	0.554–1.000
VD3	0.688	2.095	87.5%	62.5%	0.208	0.401–0.974
Glutamate	0.656	1.21E+05	62.5%	87.5%	0.294	0.362–0.950
GABRA5	0.570	0.637	62.5%	75.0%	0.637	0.248–0.893
KCC2/NKCC1	0.578	1.508	62.5%	62.5%	0.600	0.278–0.878
GABA/Glutamate	0.797	2.30E-04	75.0%	87.5%	0.046	0.561–1.000

**Table 17:** ROC analysis of the measured parameters in the control-GABA group.

Parameters	AUC	Cut-off value	Sensitivity %	Specificity %	*p* value	95% CI
GST	0.757	0.141	66.7%	87.5%	0.075	0.511–1.000
Catalase	0.847	0.631	66.7%	100.0%	0.016	0.634–1.000
Lipid peroxides	0.597	1.370	33.3%	100.0%	0.501	0.314–0.881
GSH	0.750	6.602	100.0%	50.0%	0.083	0.504–0.996
VitC	0.542	5.985	77.8%	50.0%	0.773	0.252–0.831
EAAT2	0.958	2.171	77.8%	100.0%	0.001	0.873–1.000
KCC2	0.639	21.779	44.4%	100.0%	0.336	0.356–0.922
NKCC1	0.639	9.085	44.4%	100.0%	0.336	0.360–0.917
GABA	0.750	55.545	55.6%	100.0%	0.083	0.499–1.000
VD3	0.646	2.284	66.7%	75.0%	0.312	0.363–0.928
Glutamate	0.500	1.31E+05	44.4%	100.0%	1.000	0.192–0.808
GABRA5	0.583	0.438	44.4%	100.0%	0.564	0.292–0.875
KCC2/NKCC1	0.806	1.734	66.7%	100.0%	0.034	0.577–1.000
GABA/Glutamate	0.681	5.32E-04	55.6%	100.0%	0.211	0.407–0.954

**Table 18:** ROC analysis of the measured parameters in the control-combination group.

Parameters	AUC	Cut-off value	Sensitivity %	Specificity %	*p* value	95% CI
GST	0.732	0.151	57.1%	87.5%	0.132	0.471–0.993
Catalase	0.670	0.287	71.4%	75.0%	0.272	0.363–0.977
Lipid peroxides	0.866	2.155	100.0%	62.5%	0.018	0.682–1.000
GSH	0.580	6.511	71.4%	62.5%	0.603	0.277–0.883
VitC	0.857	9.360	85.7%	75.0%	0.021	0.665–1.000
EAAT2	0.607	6.819	71.4%	62.5%	0.487	0.305–0.909
KCC2	0.839	17.953	71.4%	87.5%	0.028	0.636–1.000
NKCC1	0.607	13.301	71.4%	75.0%	0.487	0.281–0.933
GABA	0.920	41.903	85.7%	87.5%	0.007	0.781–1.000
VD3	0.589	1.873	100.0%	25.0%	0.563	0.291–0.888
Glutamate	0.536	1.15E+05	57.1%	75.0%	0.817	0.207–0.864
GABRA5	0.679	3.99E-01	42.9%	100.0%	0.247	0.384–0.973
KCC2/NKCC1	0.518	1.260	57.1%	62.5%	0.908	0.200–0.836
GABA/Glutamate	0.893	3.76E-04	85.7%	87.5%	0.011	0.725–1.000

**Table 19:** ROC analysis of the measured parameters in the PPA-GABA group.

Parameters	AUC	Cut-off value	Sensitivity %	Specificity %	*p* value	95% CI
GST	0.723	0.161	71.4%	75.0%	0.148	0.435–1.000
Catalase	0.929	0.310	100.0%	75.0%	0.005	0.798–1.000
Lipid peroxides	0.518	2.665	100.0%	25.0%	0.908	0.215–0.821
GSH	0.786	7.226	100.0%	50.0%	0.064	0.550–1.000
VitC	0.679	6.674	100.0%	50.0%	0.247	0.393–0.965
EAAT2	0.857	4.745	85.7%	75.0%	0.021	0.659–1.000
KCC2	0.554	20.287	42.9%	100.0%	0.728	0.226–0.881
NKCC1	0.679	8.707	57.1%	100.0%	0.247	0.349–1.000
GABA	1.000	50.213	100.0%	100.0%	0.001	1.000–1.000
VD3	0.732	2.284	71.4%	75.0%	0.132	0.462–1.000
Glutamate	0.696	1.07E+05	71.4%	87.5%	0.203	0.382–1.000
GABRA5	0.527	0.760	28.6%	100.0%	0.862	0.211–0.843
KCC2/NKCC1	0.750	1.919	57.1%	100.0%	0.105	0.470–1.000
GABA/Glutamate	0.982	3.61E-04	100.0%	87.5%	0.002	0.927–1.000

**Table 20:** ROC analysis of the measured parameters in the PPA-combination group.

Parameters	AUC	Cut-off value	Sensitivity %	Specificity %	*p* value	95% CI
GST	0.509	0.178	57.1%	62.5%	0.954	0.201–0.817
Catalase	0.821	0.562	71.4%	100.0%	0.037	0.558–1.000
Lipid peroxides	0.536	2.035	57.1%	75.0%	0.817	0.214–0.857
GSH	0.991	9.827	100.0%	87.5%	0.001	0.956–1.000
VitC	0.714	6.674	100.0%	50.0%	0.165	0.445–0.984
EAAT2	1.000	2.090	100.0%	100.0%	0.001	1.000–1.000
KCC2	0.839	17.953	85.7%	87.5%	0.028	0.601–1.000
NKCC1	0.857	7.708	71.4%	100.0%	0.021	0.643–1.000
GABA	1.000	68.753	100.0%	100.0%	0.001	1.000–1.000
VD3	0.571	1.811	100.0%	25.0%	0.643	0.267–0.876
Glutamate	0.964	1.11E+05	100.0%	87.5%	0.003	0.877–1.000
GABRA5	0.598	0.576	71.4%	62.5%	0.524	0.274–0.923
KCC2/NKCC1	1.000	1.842	100.0%	100.0%	0.001	1.000–1.000
GABA/Glutamate	1.000	6.24E-04	100.0%	100.0%	0.001	1.000–1.000

### 3.6 Histopathological findings

**Figure 3:**
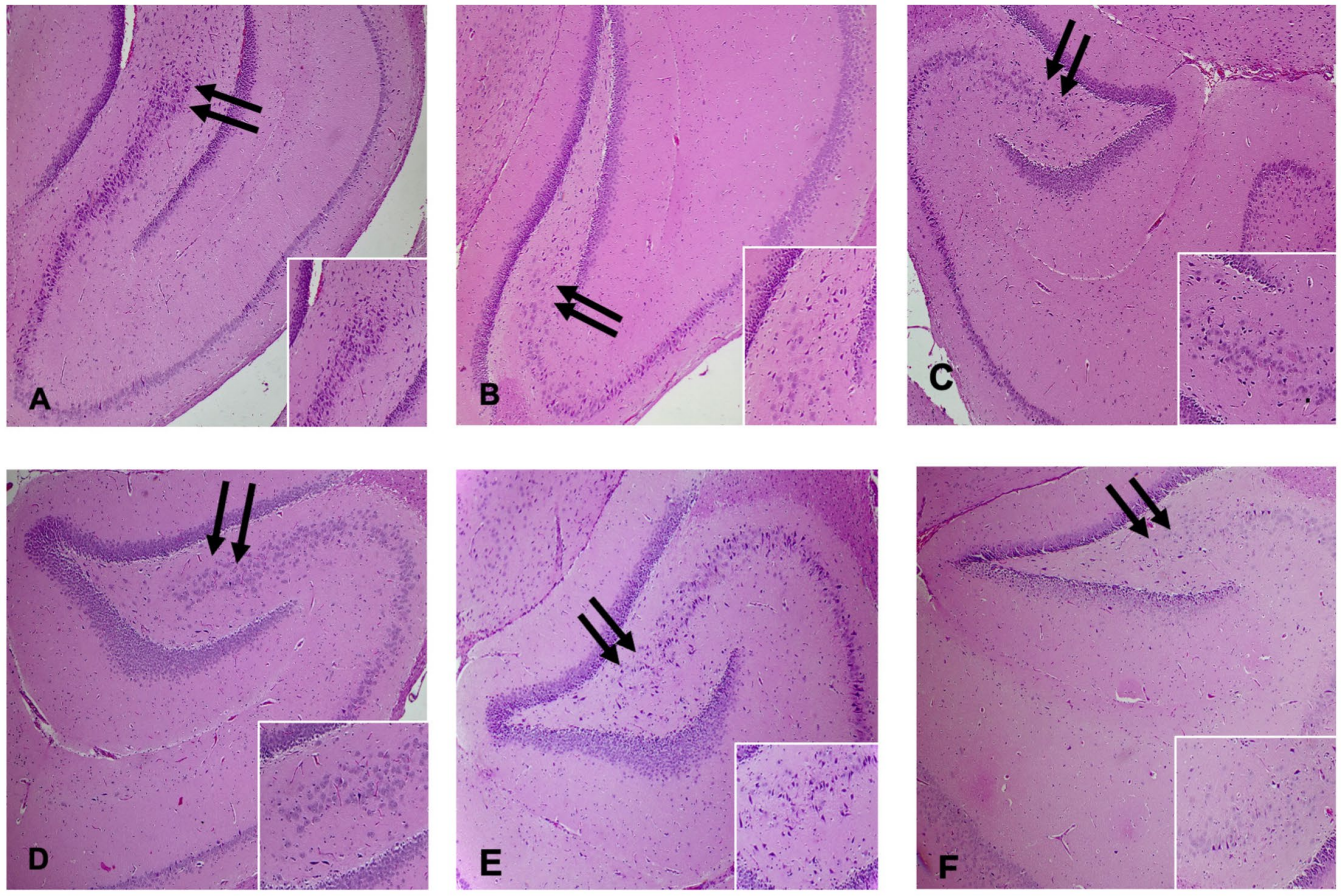
H&E-stained sections of the hippocampus with arrows pointing to the CA4 region (400X low power field). Inset: Higher magnification demonstrated the cellular density in the CA4 region of the hippocampus for each group. **(A)** Control group, **(B)** PPA-treated group, **(C)** Control-GABA group, **(D)** Control-combination group, **(E)** PPA- GABA group, and **(F)** PPA- combination group.

The Histopathology studies examined various parts of each brain sample (two rats from each group) such as the cerebrum, cerebellum, and hippocampus. Visible changes were observed in the hippocampus, as illustrated in Figure 3. In the control group, there was maintained cellular density with 5–6 cellular layers and no degenerative changes under high magnification (Figure 3A) in the CA4 region of the hippocampus. Rats in the group intoxicated with PPA (PPA-treated group) exhibited decreased cellular density and degenerative changes, including prominent eosinophilic cytoplasm and pyknotic nuclei (Figure 3B). Control-GABA group showed milder findings than PPA group, with mild degenerative changes observed (Figure 3C). Control-combination group displayed similar cellular density with no degenerative changes compared to the control group (Figure 3D). The rats receiving GABA therapy (PPA-GABA group) exhibited degenerative changes and reduced neuron density in the CA4 region, similar to PPA-treated group (Figure 3E). PPA-combination group did not show any significant changes compared to the control group (Figure 3F). The changes in cellular density and degenerative features varied across the different experimental groups, with PPA group showing the most pronounced alterations. It is worthy to note that PPA caused severe hippocampal degeneration, while combination therapy provided strong neuroprotection.

## 4 Discussion

This study highlighted the multi-faceted impact of GABA supplementation, and combination therapy (GABA, probiotics, VD3, and EAAT2 activator, *β*-lactam) on neurotransmitter balance, oxidative stress and social behavior in PPA-induced rodent model of autism. The obtained results were in line with growing evidence suggesting that an imbalance between excitatory and inhibitory neurotransmission, particularly involving glutamate and GABA, is central to ASD pathophysiology (Rubenstein and Merzenich, 2003).

PPA administration induced marked biochemical, behavioral, and histopathological changes in the rodents, modeling the E/I imbalance seen in ASD. PPA caused a significant reduction in GABA levels while increasing glutamate excitotoxicity, mimicking the neural excitation dominance observed in ASD (El-Ansary et al., 2017; Ajram et al., 2019). This imbalance directly correlates with oxidative stress, as seen in the elevated levels of lipid peroxidation and decreased antioxidant defenses (GSH, catalase), which have been previously implicated in autism-related neuroinflammation and neurodegeneration (Usui et al., 2023).

Histopathologically, PPA-treated rats exhibited hippocampal neurodegeneration, a crucial region for social and cognitive processing (Long et al., 2024). Structural and functional abnormalities in the hippocampus are frequently reported in individuals with ASD (Long et al., 2024; Etherton et al., 2011; Guo et al., 2021), and the degeneration observed in this study suggested that oxidative damage and E/I imbalance contributed to its deterioration. Individuals with ASD are reported to have elevated glutamate levels in the amygdala and hippocampus complex, supporting this excitotoxicity link (Page et al., 2006). The PPA-treated rats also exhibited impaired social interactions and increased immobility, indicating anxiety-like behavior, both common features in ASD models (Schwartzer et al., 2013; Mirza and Sharma, 2018; Alò et al., 2021). These behavioral deficits were closely tied to reduced GABA levels, which impair social processing (Parrella et al., 2024). Abruzzo et al. (2021) showed that oxidative stress disrupts chloride co-transporters in the hippocampus, contributing to E/I imbalances and behavioral abnormalities in ASD models. Overall, oxidative damage and E/I imbalance in the hippocampus appeared to drive the behavioral and social impairments observed in PPA-treated rats, mimicking ASD-like symptoms.

GABA supplementation provided moderate improvements in both neurotransmitter balance and oxidative stress markers. Biochemically, GABA levels were increased, leading to a slight reduction in glutamate levels and an improvement in the E/I balance. This restoration of GABAergic signaling helped in reducing glutamate excitotoxicity, nevertheless the improvements were modest compared to the effects observed with combination therapy. GABA supplementation slightly increased the expression of GABRA5 which enhanced inhibitory neurotransmission (Jacob, 2019), however, it was not sufficient to fully counteract the elevated glutamate levels or oxidative stress present in PPA-treated rats.

Behaviorally, GABA-treated rats showed improved social interactions compared to the PPA-treated group, suggesting that enhancing GABAergic signaling could mitigate some of the social deficits associated with ASD. This observation supports evidence from other studies, such as the work of Cochran et al. (2015), which highlighted the central role of GABAergic deficits in ASD-related social impairments. However, the improvement was moderate, emphasizing the need for more comprehensive therapeutic approaches that target both GABAergic and glutamatergic systems. For instance, studies with GABA agonists like baclofen have shown that these agents can significantly ameliorate symptoms such as hyperactivity, impaired social interaction, and spatial memory deficits in gene-altered mice (Han et al., 2012; Han et al., 2014). In mouse models of E/I dysfunction, GABA_B receptor agonists like arbaclofen have been shown to restore aspects of the E/I balance, resulting in improved behavioral deficits (Gandal et al., 2012).

Histopathologically, while GABA supplementation helped in preserving some aspects of hippocampal structure, significant neurodegeneration and reduced neuron density remained, particularly in the CA4 region, similar to what was observed in the PPA-treated group. This aligns with the moderate behavioral improvements seen in social interactions, suggesting that oxidative stress and excitatory overactivity continued to drive damage despite partial restoration of GABAergic function. Similar outcomes have been observed in the BTBR mouse model of autism, where the hippocampal commissure is smaller than usual, Silverman et al. (2015) demonstrated that enhancing GABA signaling through arbaclofen improved sociability and reduced neural deficits. Additionally, GABRA5 receptors play a crucial role in maintaining inhibitory control in the hippocampus, and modulating these receptors can help rebalance E/I dynamics. Targeting these receptors has been shown to improve cognitive and social behaviors in ASD models (Jacob, 2019), however in this study, the neuroprotective effects of GABA supplementation were limited.

The combination of GABA, probiotics, VD3, and *β*-lactam was significantly the most effective across all metrics such as GABA/Glutamate and KCC2/NKCC1 ratios. This multi-faceted approach restored inhibitory effect of GABA, reduced glutamate excitotoxicity, and normalized oxidative stress markers to near-control levels. This suggested that the combination therapy effectively addressed both E/I imbalance and oxidative stress, leading to the most substantial improvements in social behavior and neural integrity (Salim, 2017; Li, 2022).

The behavioral and histopathological outcomes observed in the combination therapy group were highly compelling. Social interaction in the treated animals was restored to near-normal levels, and immobility was significantly reduced, indicating improvements in anxiety-like behavior. Histopathologically, the combination therapy provided substantial neuroprotection, nearly eliminating the neurodegenerative changes typically seen in the hippocampus of PPA-treated rats. These results were in line with previous studies which showed that multi-faceted approaches addressing both glutamate excitotoxicity and E/I imbalances can lead to significant behavioral recovery and preservation of neural integrity in ASD models.

Oral intake of GABA has been shown to impact the brain, as evidenced by changes in EEG readings and cognitive performance improvements (Almutairi et al., 2024). This is further supported by the findings of Tabouy et al. (2018) who demonstrated that treatment with *L. reuteri* decreased repetitive behaviors and increased GABA receptor gene expression (GABRA1, GABRA3, and GABRB1) as well as protein levels (GABRA1) in the hippocampus and prefrontal cortex (PFC) of *Shank3* mutant mice, a model of ASD. Bravo et al. (2011) also highlighted how treatment with *Lactobacillus* regulated emotional behavior and central GABA receptor expression via the vagus nerve, which connects the brain and gut, suggesting the potential for probiotics to modulate the gut-brain axis in ASD treatment.

Moreover, probiotics that stimulate inhibitory neurotransmission by increasing GABA levels and its receptors may contribute to restore the E/I balance, which is often disrupted in ASD. This restoration could improve social interactions, as previously demonstrated in a study of El-Ansary et al. (2018). Additionally, it normalizes the PPA-induced increase in brain-derived neurotrophic factor (BDNF) transcript levels in the hippocampus, further supporting neuroplasticity and cognitive recovery (Abuaish et al., 2021).

GABA/Glutamate and KCC2/NKCC1 ratios in the brain tissue were significantly higher in PPA-combination group compared to all groups. This supported the hypothesis of El-Ansary (2020) which suggested that the increased expression of KCC2/NKCC1 lead to the most an inhibitory affect of GABA. These findings strongly supported that the alteration of KCC2 function in neurodevelopmental disorders leads to increased excitatory effects of GABA and abnormalities in social behavior and memory retention (Moore et al., 2019), a disruption that can be corrected through combination therapy in autism models. GABA_A receptors activation plays a crucial role in regulating KCC2 activity by stabilizing KCC2 at the plasma membrane, while inhibition of GABA_A receptors leads to decreased KCC2 stability through increased diffusion and endocytosis (Heubl et al., 2017). This mechanism is vital for maintaining chloride homeostasis and ensuring effective inhibitory neurotransmission in hippocampal circuits, which are essential for learning, memory, and overall neural regulation (Hamze et al., 2024). In the hippocampus, KCC2 mRNA levels are closely linked to GABA function, with overexpression of KCC2 shifting GABA’s action from excitatory to inhibitory, which helps maintaining the E/I balance (Ben-Ari, 2002). Proper timing of this GABA switch, mediated by KCC2, is crucial for brain development, and exposure to early-life stress can delay this switch, causing behavioral abnormalities (Furukawa et al., 2017). For instance, Tsukahara et al. (2016) found that repeated stress in mice reduced novel object recognition ability and social behavior, which was associated with decreased KCC2 levels in the hippocampus, highlighting the importance of KCC2 in maintaining healthy cognitive and social function.

VD3 has been closely linked to neuroprotection, primarily through its ability to reduce neuroinflammation and enhance GABA synthesis. Studies have shown that long-term treatment with vitamin D promotes GABA synthesis in crucial brain areas such as the prefrontal cortex, anterior cingulate cortex, and hippocampus (Staal et al., 2012). In addition to boosting GABA production, vitamin D exerts a potent antioxidant effect, inhibiting the production of nitric oxide synthase and upregulating glutathione levels, which helps reduce glial cell activation and neuroinflammation (DeLuca, 2016). These anti-inflammatory and neuroprotective properties are enhanced by vitamin D’s ability to upregulate antioxidant-related genes, including those encoding superoxide dismutase and thioredoxin reductase (Halicka et al., 2012). In ASD models, vitamin D deficiency has been linked to worsened ASD-like behaviors and lower serum vitamin D levels, which negatively impact brain development. In contrast, interventions using vitamin D have shown significant improvements in both the growth and behavior of ASD rats (Cannell, 2017). Furthermore, research indicates that low vitamin D levels are associated with reduced hippocampal volume and impaired structural connectivity, which can contribute to cognitive deficits seen in neurodegenerative disorders (Al-Amin et al., 2019).

While the *β*-lactam component was intended to increase EAAT2 expression and reduce glutamate toxicity (Takahashi et al., 2015), EAAT2 levels were surprisingly lower (17.51% of control) in the PPA-combination group. This decrease could be a compensatory response to the reduced extracellular glutamate levels resulting from the combination therapy. Research shows that glutamate transporters like EAAT2 dynamically adjust their density in response to changes in ambient glutamate (Borisova, 2016). In this context, the reduction in EAAT2 may help balance signaling by limiting glutamate uptake under conditions of low extracellular glutamate. Similar adaptive mechanisms have been observed in other neurological conditions, where reduced EAAT2 levels in certain brain regions appear to help maintain E/I balance (O'Donovan et al., 2017; Ryan et al., 2021).

Additionally, studies have shown that β-lactam antibiotics are associated with improved behavior and neuronal integrity. Research by Ho et al. (2014) and Feng et al. (2014) found that β-lactam antibiotics alleviated memory and cognitive impairments while significantly reducing cellular loss in the hippocampus, a region critical for learning and memory. These findings suggest that EAAT2 regulation is a complex process, adapting to the neurochemical environment influenced by therapies like β-lactam antibiotics. Future research is needed to further understand how EAAT2 dynamics contribute to managing excitotoxicity and maintaining neurotransmitter balance.

This study painted a cohesive picture of how PPA-induced disruptions in neurotransmitter balance and oxidative stress led to the behavioral and neural abnormalities seen in autism models. GABA supplementation alone partially alleviated these issues but was insufficient to fully restore function, highlighting the complexity of the disorder. In contrast, the combination therapy targeted multiple pathological mechanisms, offering a more comprehensive solution that significantly improved both neurochemical and behavioral outcomes.

## 5 Limitation

Since the mechanism causing the sex-biased prevalence of ASDs is still poorly understood, animal models of the disorder could help us better understand how biological sex influences these disorders. Regretfully, the use of only male rats in this study and the lack of female rats may be viewed as limitations. Future research on both sexes is necessary.

## 6 Conclusion

This study demonstrated that GABA supplementation and combined nutritional therapies (GABA, probiotics, VD3, and β-lactam) offer significant neuroprotective effects in a PPA-induced rodent model of autism. While GABA alone showed improvements in some oxidative stress markers and partial restoration of excitatory-inhibitory balance, the combination therapy was more effective in reversing neurochemical and behavioral deficits associated with PPA-induced neurotoxicity. The combination therapy significantly restored GABA and glutamate levels improved the GABA/Glutamate and KCC2/NKCC1 ratios, and enhanced antioxidant defenses, particularly GSH levels. Histopathological analysis confirmed that the combination treatment preserved hippocampal cellular integrity, which was severely compromised in the PPA-treated group. These findings suggested that targeting multiple pathways, including GABAergic modulation, oxidative stress reduction, and glutamate transporter enhancement could be a promising therapeutic strategy for alleviating autism symptoms. Further research is needed to explore the long-term efficacy and safety of these interventions in clinical settings.

## Data Availability

The original contributions presented in the study are included in the article/Supplementary material, further inquiries can be directed to the corresponding author.
