# Supplementary material for: Impact of GABA and nutritional supplements on neurochemical biomarkers in autism: a PPA rodent model study
Source: Front Mol Neurosci. 2025 Mar 18;18:1553438. doi: 10.3389/fnmol.2025.1553438 (PMC11959029; doi:10.3389/fnmol.2025.1553438)
Supplement: Supplementary file 1 [file Data_Sheet_1.pdf]

Supplementary Material

Supplementary Figures

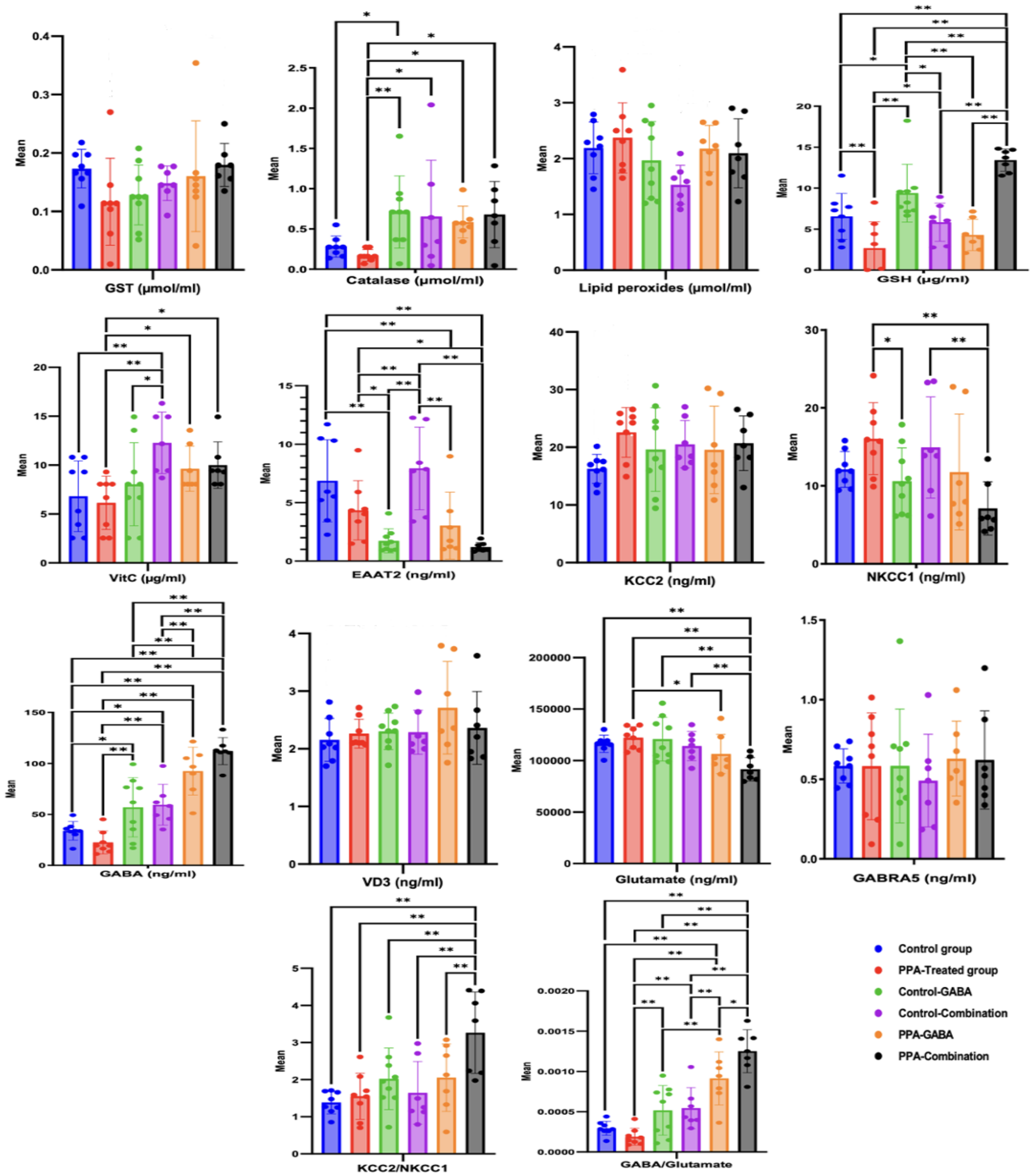

Supplementary Figure 1. Mean values (± SD) for various biochemical markers across six experimental groups: Control group (n=8), PPA-Treated group (n=8), Control-GABA group (n=9), Control-Combination group (n=7), PPA-GABA group (n=7), and PPA-Combination group (n=7). Pairwise comparisons between all groups are shown for the following parameters: GST, Catalase, Lipid peroxides, GSH, Vitamin C, EAAT2, KCC2, NKCC1, GABA, VD3, Glutamate, GABRA5, KCC2/NKCC1 ratio, and GABA/Glutamate ratio. Significant one-way ANOVA was followed by multiple comparisons by LSD or Kruskal-Wallis followed by Mann-Whitney tests, with significance indicated at *$p < 0.05$, **$p < 0.01$, and ***$p < 0.001$.

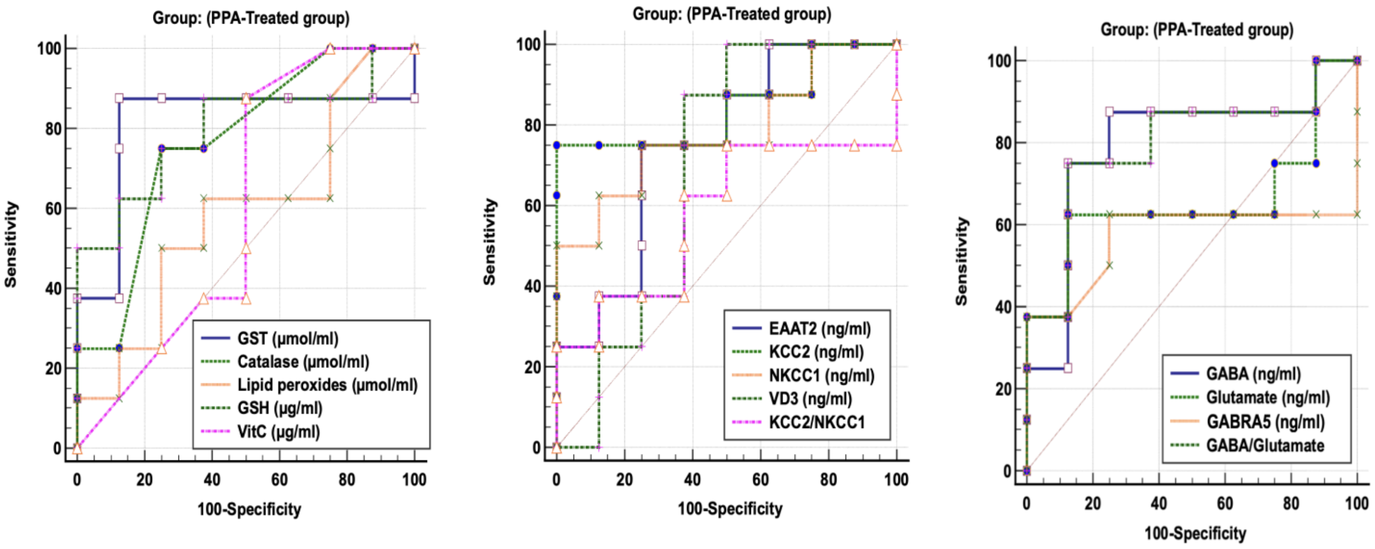

Supplementary Figure 2. ROC Curves of the measured parameters in PPA-Treated group.

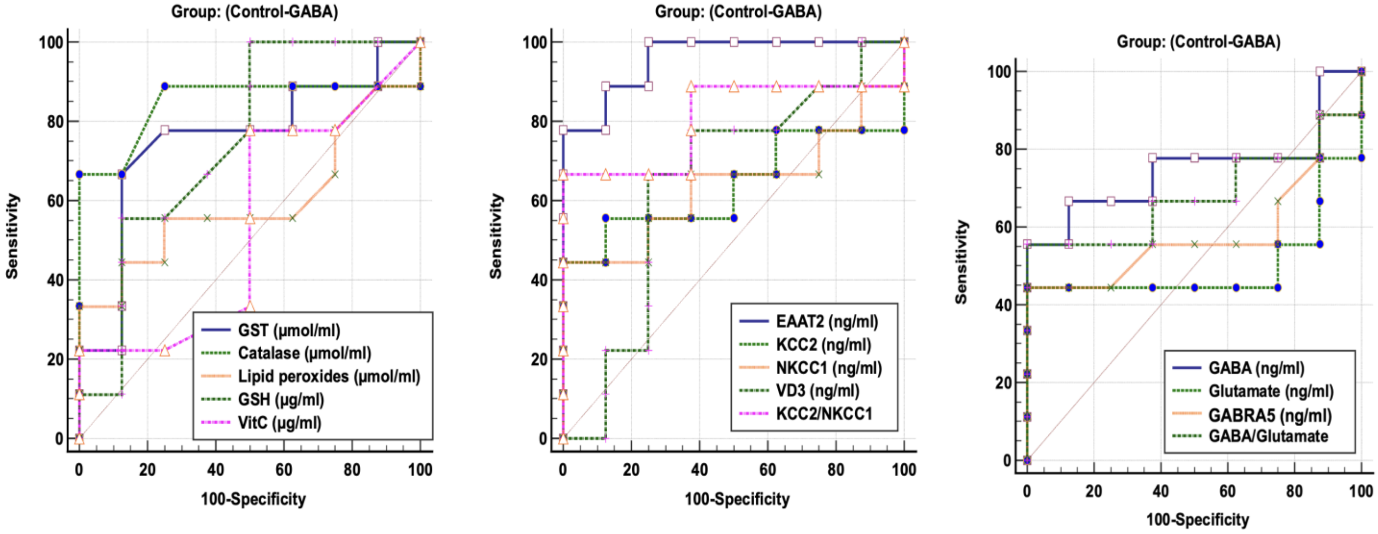

Supplementary Figure 3. ROC curves of the measured parameters in the Control-GABA group.

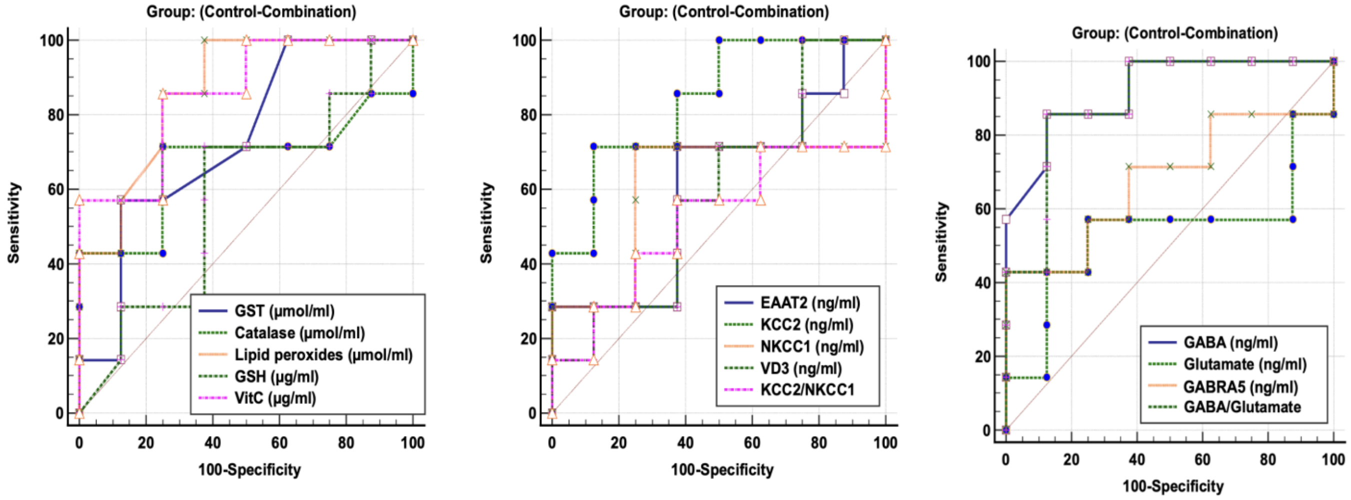

Supplementary Figure 4. ROC Curves of the measured parameters in the Control-Combination group.

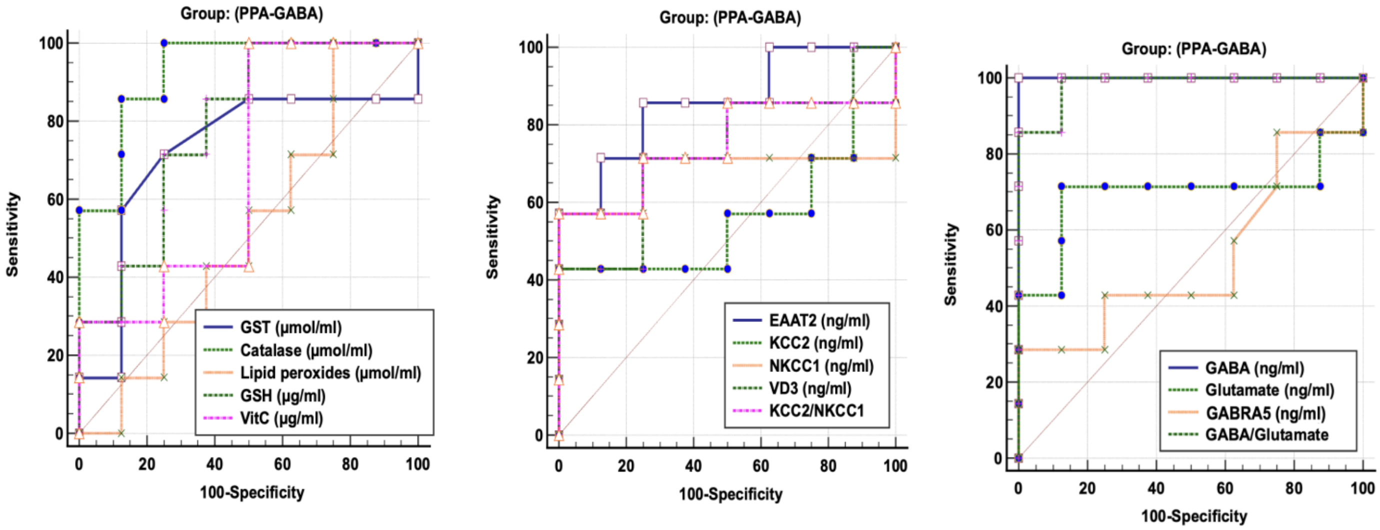

Supplementary Figure 5. ROC curves of the measured parameters in the PPA-GABA group.

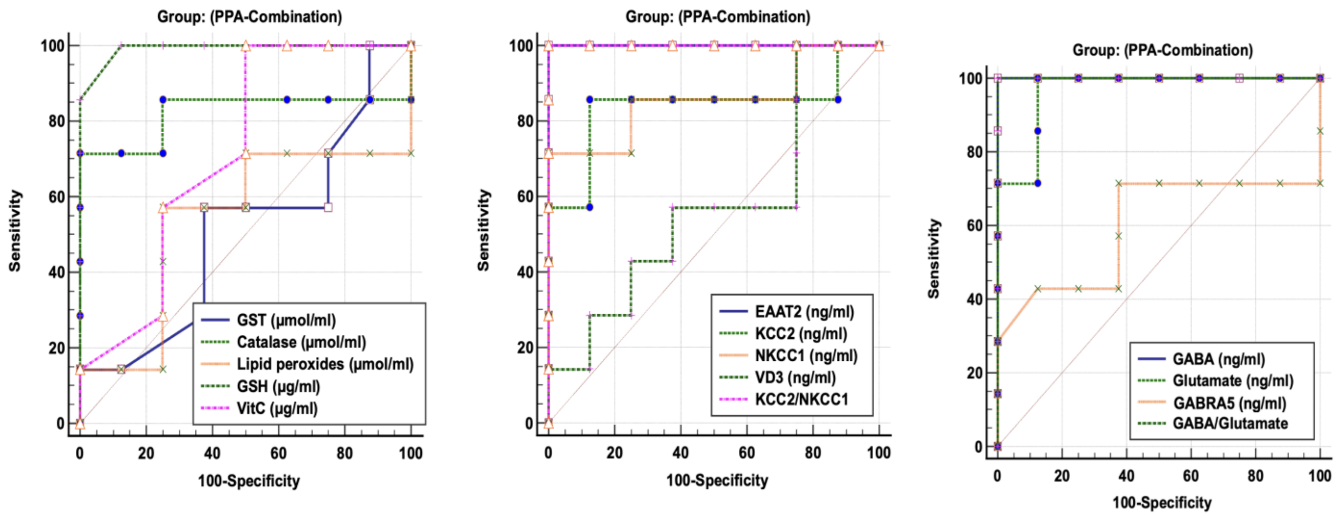

Supplementary Figure 6. ROC curves of the measured parameters in the PPA-Combination group.
